# The Cpf1 CRISPR-Cas protein expands genome-editing tools

**DOI:** 10.1186/s13059-015-0824-9

**Published:** 2015-11-17

**Authors:** Robert D. Fagerlund, Raymond H. J. Staals, Peter C. Fineran

**Affiliations:** Department of Microbiology and Immunology, University of Otago, PO Box 56, Dunedin, 9054 New Zealand

**Keywords:** Cpf1, CRISPR-Cas, Cas9, Genome editing, Bacteriophage resistance

## Abstract

CRISPR-Cas systems have immense biotechnological utility. A recent study reveals the potential of the Cpf1 nuclease to complement and extend the existing CRISPR-Cas9 genome-editing tools.

## Phage resistance provides ‘biotech bounty’

In the 100 years since their discovery, bacteriophages have significantly shaped our understanding of fundamental biological processes, including those pertinent to the central dogma of molecular biology, and have ‘gifted’ us their enzymes (including T4 ligase and T7 RNA polymerase) as biotechnological tools [1]. In addition, studies of phage–bacterium interactions have uncovered a diverse range of resistance mechanisms [2], which have provided further reagents, including restriction enzymes and CRISPR-Cas (clustered regularly interspaced short palindromic repeat-CRISPR associated) systems [1–3]. The CRISPR-Cas systems, in particular the Cas9 protein, have captured the imagination of researchers because they provide highly programmable systems that have a wide array of molecular biology applications [3]. In a recent *Cell* article, the Zhang laboratory and their collaborators have added a new Cas protein, Cpf1, to this biotechnological arsenal [4].

CRISPR-Cas systems endow prokaryotes with an adaptive immunity against phages and other mobile genetic elements, such as plasmids [1–3]. These systems are widespread, found in half of bacteria and most archaea, and they are evolutionarily diverse [5]. Makarova and colleagues recently refined the classification of CRISPR-Cas systems and proposed two major classes incorporating five types of system, which are further categorized into subtypes [5]. Of these five types, only three had been studied in detail (the class 1 types I and III and the class 2 type II (Cas9) systems) [5]. CRISPR-Cas systems function in three steps. First, ‘adaptation’ involves the addition of invader DNA as a ‘spacer’ into the CRISPR array — the ‘memory’. Second, during ‘expression’ the CRISPR array(s) are transcribed and processed to form guide CRISPR RNAs (crRNAs) consisting of repeat and spacer sequences. Finally, in ‘interference’ the crRNA in complex with Cas proteins uses the spacer to recognize a sequence termed a protospacer and degrades the target nucleic acids. The characterization of Cpf1 demonstrates for the first time that the type V class 2 systems are functional CRISPR-Cas systems [4].

**Fig. 1:**
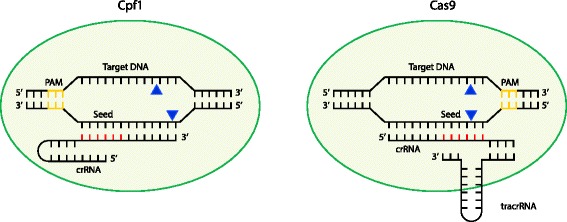
Schematic comparison of target recognition and degradation by Cpf1 and Cas9. An R-loop is formed as a result of protospacer adjacent motif (*PAM*) recognition (*yellow*), and subsequent base-pairing interactions occur between the CRISPR RNA (*crRNA*) and its cognate target sequence. Note that the guide RNA in Cas9 is an RNA duplex involving crRNA and trans-activating CRISPR RNA (*tracrRNA*), whereas Cpf1 uses a single crRNA. Upon sufficient complementarity in the seed region (*red*), Cpf1 and Cas9 nucleases will make two single-stranded cuts (*blue triangles*) resulting in a double-stranded break. DNA and crRNA lengths and cleavage positions are schematic only and are not drawn to scale

The type II systems, consisting of the Cas9 interference protein and two RNAs (a trans-activating crRNA (tracrRNA) and the crRNA) are ‘streamlined’ relative to the multi-protein type I and III interference complexes [6]. This simplicity, and the ability to replace the two RNAs with an engineered single guide RNA (sgRNA), led the Charpentier and Doudna groups to propose Cas9 for genome editing [6]. In the past three years, immense academic and commercial interest has pushed the technology from a concept to a widely used molecular biology tool [3]. Cas9 can be RNA-guided to target DNA in a sequence-specific manner and catalyzes double-stranded breaks (DSBs) (Fig. 1). The blunt DSBs are formed by two separate nicks, catalyzed by the RuvC- and HNH-like domains present in Cas9, instigating host-mediated DNA repair that can be exploited to facilitate mutant generation [3]. In addition, catalytically inactive Cas9 can be localized without DNA cleavage for multiple applications, such as the repression or activation of gene expression or imaging [3]. The broad applicability of CRISPR-Cas9 has allowed genetic manipulation in a huge variety of organisms, including viruses, bacteria and eukaryotes. Despite rapid advances, this nascent technology has scope for improvement, and Cpf1 may offer further progress [4].

## Cpf1: a genome-editing alternative

Schunder and colleagues identified the *cpf1* and *cas* genes, with their associated CRISPR arrays, in *Francisella* spp. and suggested that they were functional due to the presence of spacers that are similar to prophages [7]. Makarova et al. [5] subsequently proposed a new classification for CRISPR-Cas systems that included the type V CRISPR-Cas systems, which are characterized by the Cpf1 ‘signature’ protein. Zetsche et al. [4] tested the function of CPf1 by cloning the *Francisella novicida cpf1* (FnCpf1), cas genes operon and CRSIPR array into *Escherichia coli*. During interference in type I and II CRISPR-Cas systems, target interrogation is initiated by searching for a protospacer adjacent motif (PAM), after which target recognition is completed by base-pairing between the crRNA and the protospacer. By screening for interference against a plasmid library containing variable PAMs, Zetsche et al. [4] were able to identify the PAM requirements for FnCpf1 (5′-TTN-3′ and 5′-CTA-3′ on the displaced strand), and in doing so provided the first evidence that type V systems are genuine CRISPR-Cas systems. Fifteen other Cpf1-family proteins displayed a similar 5′-TTN-3′ or 5′-TTTN-3′ PAM selectivity [4]. Surprisingly, the PAM for Cpf1 is on the opposite end of the protospacer when compared with that for Cas9, yet is similar to that for the class 1, type I systems. Furthermore, most Cas9 proteins have a G-rich PAM preference; the PAM for the well-characterized *Streptococcus pyogenes* Cas9 (SpCas9) is 5′-NGG-3′ [6]. Although PAM selectivity limits interference targets, the PAM repertoire can be expanded by utilizing Cas9 orthologs [8, 9] or by engineering Cas9 variants to recognize other PAMs and reduce off-target cleavage [10]. Cpf1 further extends the potential targets utilized by existing Cas9 proteins and might be useful for manipulation of A/T-rich genomes [4].

A major difference between Cas9 and Cpf1 proteins is that Cpf1 does not utilize tracrRNA, and thus requires only a crRNA (Fig. 1). The FnCpf1 crRNAs are 42–44 nucleotides long (19-nucleotide repeat and 23–25-nucleotide spacer) and contain a single stem-loop, which tolerates sequence changes that retain secondary structure [4]. The Cpf1 crRNAs are significantly shorter than the ~100-nucleotide engineered sgRNAs required by Cas9, and thereby offering cheaper and simpler guide RNA production. Furthermore, the different sgRNA and crRNA requirements of Cas9 and Cpf1 will allow both systems to be combined when multiplexing of different targets is desired — for example, when genome editing is combined with gene regulation. Multiplexing is possible using orthogonal Cas9s that have different sgRNA sequence specificities, and Cpf1 will expand this potential [8, 9].

For efficient interference by Cpf1, the spacer-encoded portion of the crRNA requires a minimum of 18 nucleotides and a seed sequence in the first ~5 nucleotides of the 5′ end of the spacer. Seed sequences are always present adjacent to the PAM; therefore, in Cpf1 the seed sequence is at the opposite end of the protospacer to that in Cas9. Although both Cas9 and Cpf1 make DSBs, Cas9 uses its RuvC- and HNH-like domains to make blunt-ended cuts within the seed, whereas Cpf1 uses a RuvC-like domain to produce staggered cuts outside of the seed (Fig. 1) [4]. As discussed below, these differences have significant implications for the biotechnological application of Cpf1.

Zetsche and colleagues tested whether Cpf1 could perform genome editing in human cells [4]. Eight different Cpf1 proteins were tested and all cleaved DNA in vitro, but only two proteins from the *Acidaminococcus* and *Lachnospiraceae* genera yielded detectable insertion/deletion (indel) mutations in vivo at levels similar to those produced by SpCas9. It is proposed that the differences in the cleavage mechanisms of Cpf1 compared to Cas9 offers the biggest potential benefit for genome editing. Two major mechanisms are used during genome editing to repair DSBs: non-homologous end-joining (NHEJ) and homology-directed repair (HDR). In the case of Cas9, error-prone NHEJ is dominant and results in indels that will disrupt the Cas9 target site and impede HDR. Because Cpf1 makes staggered cuts away from the critical seed region, NHEJ will not disrupt the target site, therefore ensuring that Cpf1 can continue to cut the same site until the desired HDR recombination event has taken place. This potential benefit requires confirmation in future studies.

## Type V CRISPR-Cas systems

In addition to offering potential advances in genome editing, Cpf1 has begun to provide fascinating insights and questions regarding the biology of type V CRISPR-Cas systems. Interestingly, the type V (class 2) systems share common features with class 1 systems. There are currently no data on spacer acquisition by type V systems, but the type V Cas1 and Cas2 (and Cas4) adaptation proteins are more evolutionarily related to type I and III (class 1) proteins [5], suggesting that spacer acquisition by type V systems has more similarities to that in class 1 systems than to that in class 2 systems. For crRNA biogenesis, type II systems need Cas9, tracrRNA and host RNaseIII. RNA sequencing in *F. novicida* and in *E. coli* containing only Cpf1 and the CRISPR array revealed similar crRNA profiles [4]. Although it is possible that a host ribonuclease is involved, crRNA generation seems to require only Cpf1. Interference by type V systems also has features that are reminiscent of type I systems. Indeed, the PAM and seed are in the same location as in type I systems and the PAM is similar to the most common one in *E. coli* type I-E (5′-TTN-3′ compared with 5′-TTC-3′). A full in vivo seed analysis is required to understand interference specificity, which will be important for genome-editing applications.

Within the recent CRISPR-Cas classification [5], the only systems that have not been characterized experimentally are type IV members of class 1. Type IV systems are likely to form multi-protein complexes, but they do not appear to be associated with *cas1* and *cas2* or with CRISPR arrays, raising intriguing questions about their mode of action. From this recent study from the Zhang laboratory, it is evident that Cpf1 offers new avenues for biotechnological exploitation. Undoubtedly, the analysis of other CRISPR-Cas systems will continue to provide further tools for molecular biology.

## Abbreviations

Cas: CRISPR-associated
CRISPR: clustered regularly interspaced short palindromic repeat
crRNA: CRISPR RNA
DSB: double-stranded break
HDR: homology-directed repair
NHEJ: non-homologous end-joining
PAM: protospacer adjacent motif
sgRNA: single guide RNA
tracrRNA: trans-activating crRNA
